# Supplementary material for: All-day fresh water harvesting by microstructured hydrogel membranes
Source: Nat Commun. 2021 May 14;12:2797. doi: 10.1038/s41467-021-23174-0 (PMC8121874; doi:10.1038/s41467-021-23174-0)
Supplement: Supplementary file 1 — Supplementary Information [file 41467_2021_23174_MOESM1_ESM.pdf]

Supplementary Information for

All-day Fresh Water Harvesting by Microstructured Hydrogel Membranes

Ye Shi[1,*], Ognjen Ilic[1,2], Harry A. Atwater[1] & Julia R. Greer[1,*]

1. Division of Engineering and Applied Science, California Institute of Technology, Pasadena, CA, USA

2. Department of Mechanical Engineering, University of Minnesota, Minneapolis, MN, USA

[*] *yeshi119@utexas.edu, jrgreer@caltech.edu*

S1 Supplementary Methods

S1.1 CAD design of membranes with micro-tree, cone, and cylinder array by Solidworks

The micro-structured gel membranes are initially designed and drawn by Solidworks software. Supplementary Figure 1a shows the blueprints of membrane with micro-tree array in 45 degree top, side, and top views. The height of tree trunk is 5 mm and its bottom diameter is 1 mm. 9 smaller cones with same conicity are distributed uniformly as branches at 1/3, 1/2, and 2/3 height of trunk with 45 degree tilted. 100

these micro-trees are hexagonally planted on a 1.5-mm thick supporting layer and the distance (center to center) between two adjacent trees is 2.5 mm. The gel thickness has been demonstrated to have no effects on saturated water content and water transport rate of hybrid hydrogels[1]. The relatively thick supporting layer used is to maintain the structure integrity of hydrogels during fabrication and processing.

The cones have exactly same size with micro-tree' trunk, with height of 5 mm and bottom diameter of 1 mm (Supplementary Figure 1b). 100 these micro-cones are hexagonally distributed on the supporting layer with inter-distance (center to center) of 2.5 mm. The hexagonal arrangement is beneficial for fog collection because with the staggered arrangement each cone is wholly wrapped by the flow stream and water drops can not only deposit on the windward side but also on the leeward side of the cones[2]. The cylinders have height of 5 mm and diameter of 1 mm (Supplementary Figure 1c). 100 these micro-cylinders are hexagonally distributed on the supporting layer with inter-distance (center to center) of 2.5 mm.

The projected area of supporting layer is designed to be slightly larger than that of micro-structures array, thus facilitating mold assisted fabrication. The supporting layer is cut to fit the area of array for final PVA/PPy gel membranes during all tests. It is also worthy to notice that PVA/PPy gels shrinks a little during cycles of freeze-thaw processing. The size of final gel structures is ~80% of the size in CAD design (in one dimension). For examples, the height of PVA/PPy gel micro-tree is ~4 mm and its bottom diameter is ~0.8 mm. The ratio of total surface areas for membranes with flat surface, cone array, cylinder array and micro-tree array is 1:1.8:2.4:3.5, respectively.

S1.2 Set-up of fog collection test in lab

To test the fog collection ability of microstructured PVA/PPy gels, a hydrogel membrane sample with 4 cm^2 projected area is placed with a inclined angle θ to the horizontal surface, meanwhile, a sustained fog flow generated by ultrasonic humidifier (LV600HH, Levoit) with a velocity of about 1 m s^{-1} is kept blowing to the surface with a tilted angle (15 degree) to the tangent direction of the membrane at room temperature (Supplementary Figure 5a)[3]. The fog flow angle is carefully selected to make sure the fog flow passes through the gel micro-structure array and minimize the influence of supporting layer. If the angle is smaller, the fog flow may directly hits the supporting layer and causes significant edge effect. When the angle is larger, we observed that part of the fog flow bounced back from the substrate and flow into the collection beaker directly.

The outlet of fog is kept 3 cm from the bottom of gel membrane. θ is tuned from 15 degree, 45 degree to 85 degree. The fog flow is just blown to the structured region and higher than the solid substrate, which helps avoid edge effect on supporting layer[3]. A beaker is placed under the gel sample to collect drained water and the amount of collected water is measured every 15 minutes. Without further clarification, the fog collection rate in this Supplementary Text is calculated based on the projected area of gel membranes. We studied the effect of inclined angle for the gravity assisted drainage and found that there was no obvious difference in resulted fog collection rate (Supplementary Figure 5b). This is mainly because the millimeter-size droplets formed by coalescence of smaller droplets from all branches have an initial speed

when they drop from the micro-trees, which facilitates their drainage. Thus θ is set as 45 degree for all fog collection tests. The room temperature for fog collection tests is 25 °c and the relative humidity in artificial fog flow is 100%.

To study the influence of fog flow velocity, fog flows with 0.5 m/s and 2 m/s speed were applied (Supplementary Figure 5c). It can be seen that as the fog flow speed increases, the time for gel micro-tree array to reach the saturated collection rate decreases but the maximum fog collection rate remains almost same. This is because it takes longer time for slower fog flow to pass through the micro-tree array and to fully wrap the whole surface of gel micro-trees. Once the array is saturated by the fog flow, the concentration of water droplets in the fog exceeds the fog collection capacity of the gel surface. The whole surface of gel membrane continuously captures droplets and transports them for collection.

S1.3 Experimental measurement of equivalent vaporization enthalpy in microstructured PVA/PPy hydrogels

To compare the water vaporization enthalpy in microstructured PVA/PPy hydrogels, we designed a control experiment to measure the vaporization enthalpy[1]. As shown in Supplementary Figure 21a, a container is set under room temperature (RT) and half of the container is filled up with supersaturated potassium carbonate solution to enable stabilized relative humidity (RH) of ~45% in the closed space. Free water and gel samples with same evaporation area are synchronously put in the closed container above potassium carbonate solution. To keep the evaporation area same, an optical

profilometer was used to measure the surface area of hydrogel, which is ~2 m^2/m^2. Then the total surface area of gel microstructures was estimated by combining the parameter from CAD file used for 3D printing. The mass change of free water and gel sample caused by water evaporation is measured every hour and corresponding equivalent evaporation enthalpy (ΔH_{equ}) is calculated based on the following equation using average evaporation amount in an hour:

$$U_{in} = \Delta H_{vap} m_0 = \Delta H_{equ} m_g$$

where U_{in} is the power input which is identical for free water and gel samples; ΔH_{vap} and m_0 are the vaporization enthalpy and average evaporation amount of free water; m_g is the average evaporation amount of gel samples.

To further prove the reduced evaporation enthalpy of water in PVA/PPy hybrid gel, differential scanning calorimetric (DSC) measurement is used for measuring the vaporization energy of pure water and water in the gel. The gel sample was placed in an open Al crucible and measured with a linear heating rate of 5 K min^{-1}, under a nitrogen flow (20 mL min^{-1}), in the temperature range from 20 to 180 °C. The effective specific heat capacity was calculated by comparing the heat flow of measured gels with that of the standard sapphire sample.

S1.4 Simulation of surface temperature distribution of PVA/PPy gel microstructures

At steady state, the net temperature and evaporation rate is determined from the energy balance between various terms: solar irradiation, convection, radiation loss,

evaporation, and loss to the water underneath. This balance can be expressed as:

$$Q_{solar} + Q_{conv} + Q_{rad} + Q_{evap} + Q_{water} = 0 \qquad \text{(S1)}$$

In our system, there a number of surfaces that are not normal to the incident light direction (z), so the energy flux due to irradiation can be expressed as $Q_{solar} = \alpha I_{solar}|\hat{n} \cdot \hat{z}|$, where α is the surface absorptivity (Fig. 4c in main text), and $I_{solar}$is the solar irradiance at Earth level. Generally, for a closed environment with controlled ambient parameters (humidity, pressure, temperature), the evaporative flux can be expressed as $Q_{evap} = H_v k(C_{sat}(T) - C_{wa})$ where H_v, k are the heat of evaporation and the mass transfer coefficient, respectively, and C_{sat}, C_{wa} are the concentration of saturated vapor and the concentration of vapor in air, respectively[4]. The saturation concentration relates to the saturation pressure p_{sat} as $C_{sat}(T) = p_{sat}(T)/RT$. The convective heat flux is expressed as $Q_{conv} = h_{conv}(T - T_a)$, where $T_a \approx 23$ ℃ is the ambient environment temperature, and h_{conv} is the convective heat transfer coefficient. The radiation loss term is proportional to emissivity of the material, the local temperature, and the background environment temperature, i.e. $Q_{rad} = \epsilon\sigma(T^4 - T_a^4)$, where σ is the Stefan-Boltzmann constant; from Kirchoff's law, we assume the emissivity of the gel structure is equal to its absorptivity, e.g. $\epsilon = \alpha$. Last, the energy flow to the underlying water is incorporated through the temperature boundary condition where the water temperature is equal to the environment temperature T_a (room temperature). We use this energy balance model to simulate and identify qualitative trends in surface temperature distribution for different morphologies (cone, cylinder, tree). In COMSOL Multiphysics, cone and cylinder

case is analyzed as a two-dimensional axisymmetric model, while the tree case is analyzed as a three-dimensional model with an illumination source incident from the top (-z direction). The energy balance of Equation (S1) is applied as the net heat flux boundary condition at "top" interfaces that are exposed to illumination/evaporation. For the edges of the boundary domain below the top interface (side walls and below surface level), we assume insulating boundary conditions (i.e. no heat flux across the boundary). In our model, we assume values for the incident solar intensity (1,000 W/m^2), heat of evaporation (~1,000 kJ/kg), convective heat transfer coefficient (~10 W/m^{2}K), and estimate $k \sim 2.2 \cdot 10^{-5}$m/s[4].

S1.5 Calculation of shape factors for different gel microstructure arrays

To get a qualitative understanding for how the inter-distance affects vapor escape in arrays with different morphology, we draw a similarity to the concept of shape factor in radiative heat transfer[5]. Shape factor is a geometrical function that depends on the size, shape, separation distance, and orientation of participating elements. The shape factor between two surfaces A and B, labelled $F_{A \to B}$, relates the proportion of radiation leaving surface A that is intercepted by surface B. We used shape factor as a geometrical characteristic to qualitatively describe the "packing density" of participating elements in our gel micro-structure arrays by showing how closely these micro-structures are packed together in one array and how much open space the array could provide for the vapor to escape. As the inter-distance of gel micro-structures on our membranes is at millimeter level, smaller shape factor indicates more open space

for vapor to escape and lower chance for generated vapor to be interfered by the adjacent micro-structures.

We numerically evaluate the shape factor (COMSOL Multiphysics) between the nearest-neighbor elements for cone, cylinder, and tree arrays. Geometrical shape factors are calculated using COMSOL Multiphysics Surface-to-Surface Radiation interface. The computational domain consists of two nearest-neighbor elements. The boundary conditions applied to the surface of the elements treat them as diffuse black-body radiators with emissivity near-unity emissivity. The shape factor, also known as the view factor, between the two elements/surfaces A1 and A2 is defined as the ratio between the diffuse energy leaving A1 and intercepted by A2 and the total diffuse energy leaving A1, that is $F_{A1-A2} = \frac{\int_{A2} \Upsilon(J_1) ds}{\int_{A1} J_1 \ ds}$, where J_1 is the radiosity of element 1, and $\Upsilon(\mathrm{J}_1)$ is the irradiation operator from the surface-to-surface radiation interface in COMSOL Multiphysics.

S1.6 Design of floating prototype for all day water collection in natural environments

We designed a floating prototype for all day water collection in natural environments, such as sea, lake, or pool. The structure was constructed from polyester thin film, cellulose-based fabric, polyurethane foam, metal wires and wood rods. The condensation structure was constructed from lightweight and cheap polyester films. The film was cut into several pieces and glued together on skeleton made by steel wires. Droplet collection was facilitated by inclined polymer film and

super-absorbable fabric wicks (Zorbs)[6]. Collected water was transported to water storage bag by fabric wicks. The PVA/PPy gel samples were held by a supporting structure which was made by polyurethane foam and nylon mesh. The wholesale materials cost of the entire floating prototype is ~ $4.

The unique feature of our floating prototype is its foldable condensation structure which enables dual mode for all day water collection. Our design can be easily replicated at home or modified and produced by factory. Smart or remote modulus can be further added to the device to enable intelligent control of water collection modes.

S2 Supplementary Figures and Tables

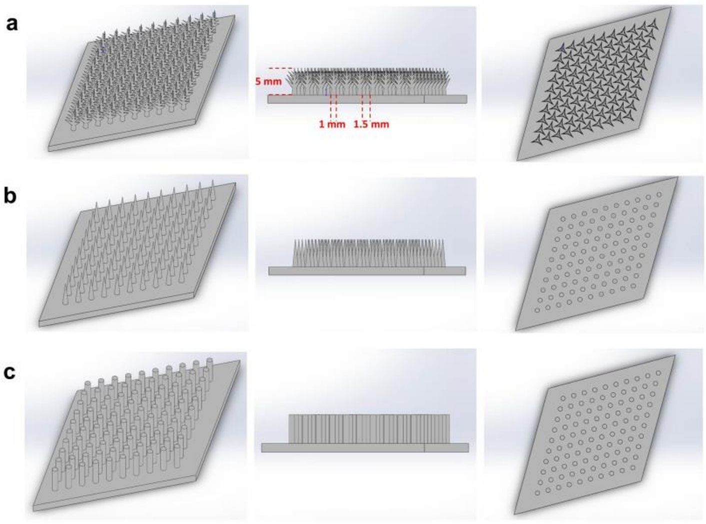

Supplementary Figure 1. CAD blueprints of membranes with (a) micro-tree, (b) cones and (c) cylinder array.

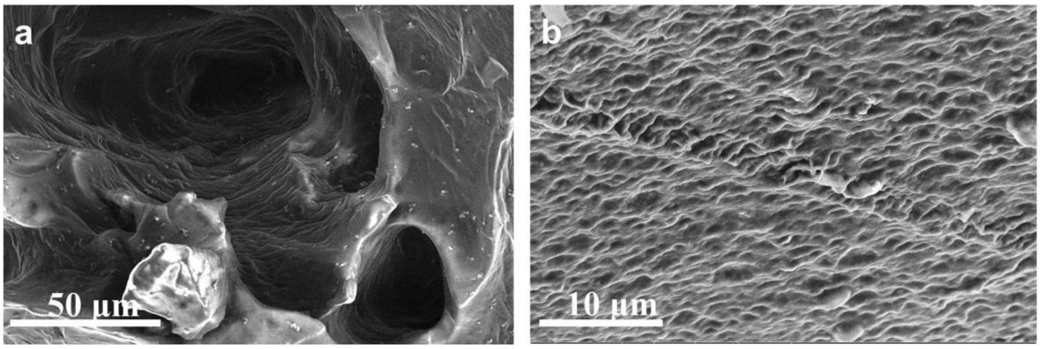

Supplementary Figure 2. SEM images of PVA/PPy hydrogels showing (a) broad internal gaps with diameters from 50 to 150 µm, which together with micro-pores enable rapid water diffusion and capillary pumping to supporting a sustained high rate vapor generation[1]. (b) the wrinkled internal surface, which indicates shrinkage of the polymeric skeleton (PVA network) during dehydration of the hydrogel.

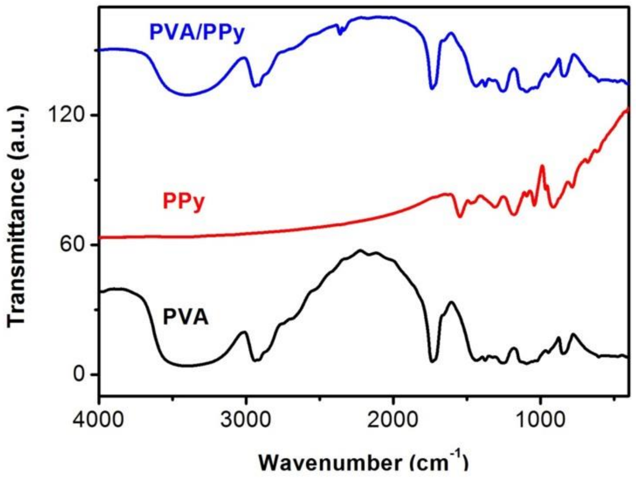

Supplementary Figure 3. FTIR spectra of PVA, PPy and PVA/PPy hybrid gels. PVA shows a characteristic peaks at 1093 cm^{-1}, which can be attributed to C–O stretching. PPy shows absorption signals at 1552 cm^{-1} and 1045 cm^{-1}, which are corresponding to the in-ring stretching of C=C bonds in the pyrrole rings and the in-plane deformation of N–H bonds, respectively. All these characteristic peaks of PVA and PPy can be found in the FTIR spectra of PVA/PPy hybrid gel, which confirms the presence of PPy in the PVA matrix. These peaks show no shifts, indicating that PPy particles are physically mixed with PVA.

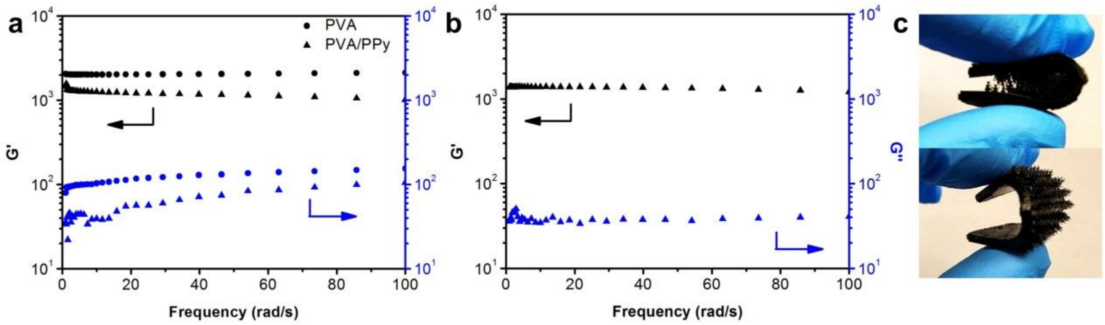

Supplementary Figure 4. (a) The storage modulus (G’) and loss modulus (G’’) of as-prepared PVA and PVA/PPy gels. Both samples exhibit solid gel behavior. The hybrid gel exhibits a ~40% lower G’ than the pure PVA gel because it has fewer crosslinking points caused by the introduction of PPy. The lower G’’ of the PVA/PPy hybrid gel indicates that the polymeric PVA chains are immobilized by the hard PPy segments. (b) The storage modulus (G’) and loss modulus (G’’) of PVA/PPy gels after ~20 month’s storage. The crosslinked network structure was well maintained after long-term storage. (c) Pictures of PVA/PPy gel micro-tree membrane being bent.

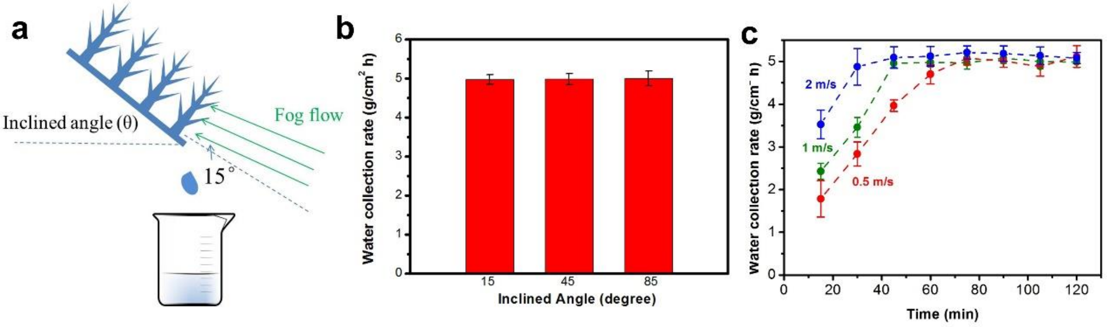

Supplementary Figure 5. (a) Schematic illustration of set-up of fog collection test in lab. (b) Fog collection rates of PVA/PPy micro-tree array with different inclined angels in lab tests. (c) Fog collection rates of PVA/PPy gel micro-tree array under different fog flow speeds.

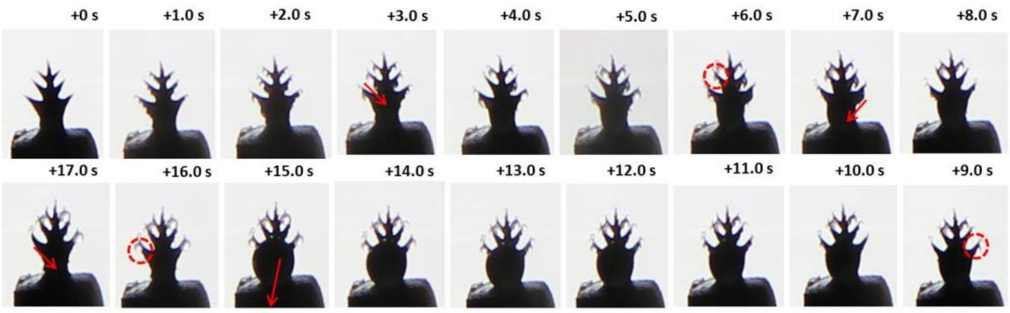

Supplementary Figure 6. Photos showing fog collection behavior of one PVA/PPy gel micro-tree.

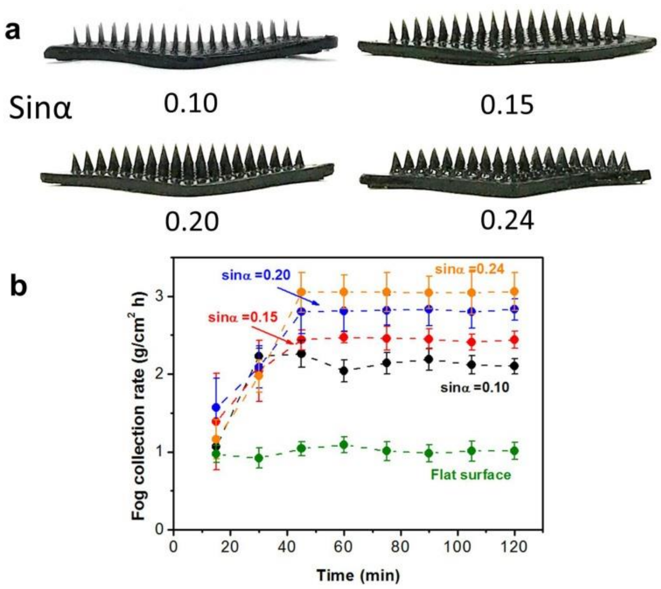

Supplementary Figure 7. (a) Photos of PVA/PPy gel cone arrays with different apex angle. (b) Fog collection rates of PVA/PPy gel cone arrays with different apex angle in fog harvesting tests. After normalizing the fog collection rates by total surface area, the fog collection ability of different cone arrays improves as the apex angle decreases, indicating faster directional movement of droplets on cones with smaller apex angle. These results indicate that while the surface area is maintained, the fog collection ability of conical gel structures can be improved by decreasing their apex angle.

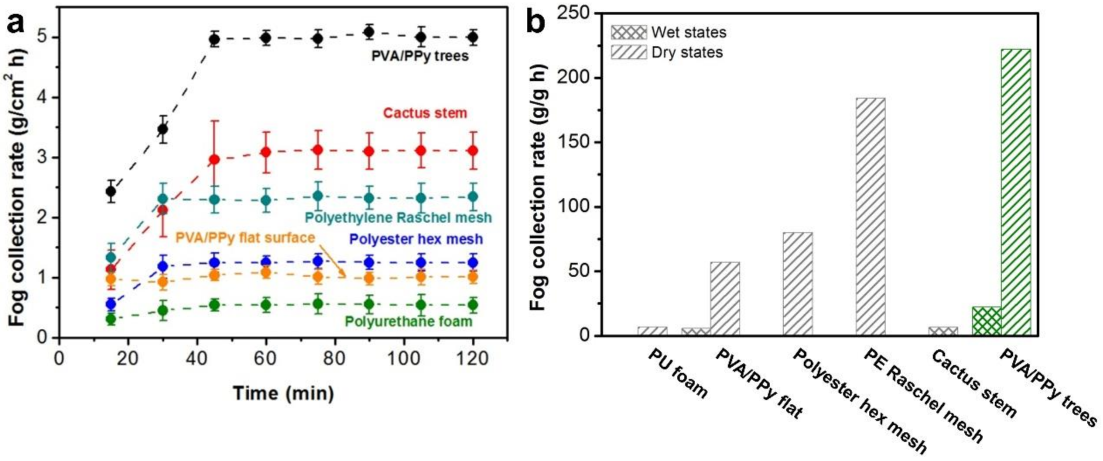

Supplementary Figure 8. (a) Fog collection rates of different materials under equivalent testing conditions. All the tested materials are cut into same diamond shape as PVA/PPy gel membrane and tested under same experimental conditions. The fog collection rates are calculated based on the projected area of these membranes. For porous Raschel mesh (double layered, 35% shading) and hex mesh (double layered, 50% shading), the fog collection rates are calculated based on their effective area (area of pores is excluded). (b) Fog collection rates of different materials calculated based on their mass in wet and dry states.

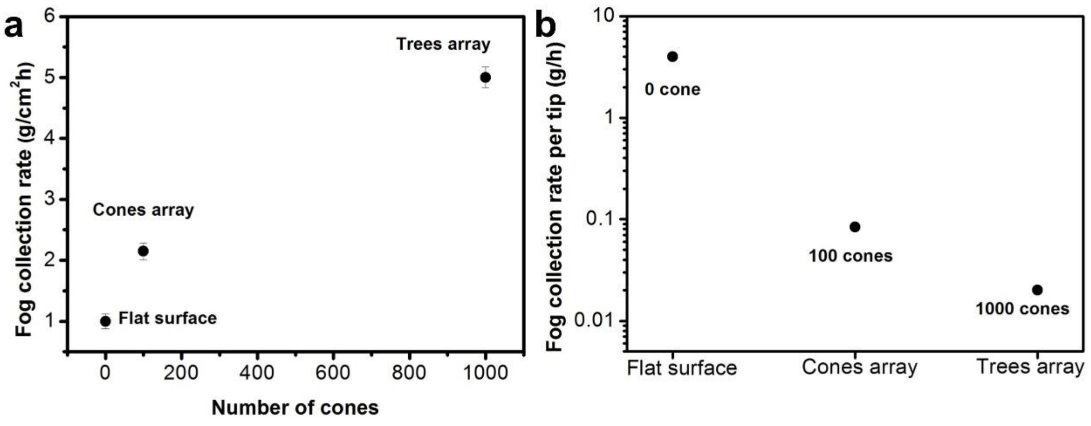

Supplementary Figure 9. (a) Fog collection rates of microstructured PVA/PPy gel membranes along with their number of cones. The droplets formed on cone tips sit for much longer time than droplets at other locations. The contribution from each cone are different in three different structures. (b) Fog collection rates normalized by number of cones for different microstructured PVA/PPy gel membranes, which indicate that the number of cones may not be a determining factor for fog collection rate.

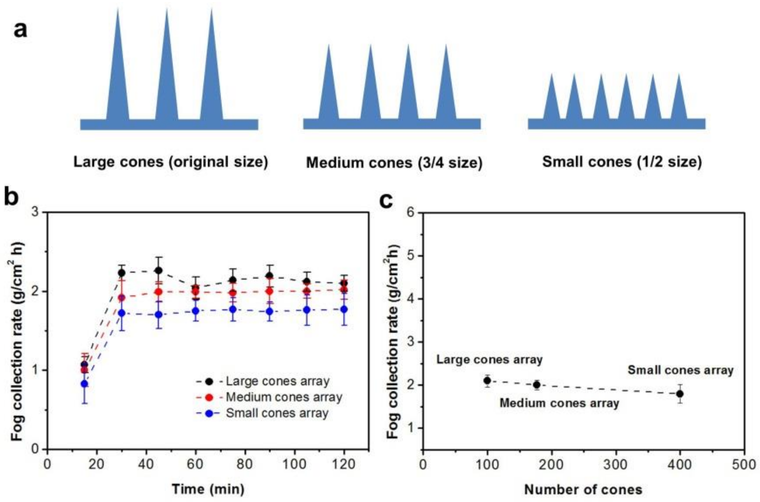

Supplementary Figure 10. (a) Schematic illustration of cone arrays with changed size (side view). The dimension (both height and bottom diameter) and inter-distance of cones are 3/4 (medium cones) and 1/2 (small cones) of original ones. (b) Fog collection rates of cone arrays with different sizes during tests. (c) Fog collection rates of cone arrays with different size and their number of cones. All these evidence indicate that the droplets deposited on the cone tips contribute little to the fog collection ability of cone-based structures.

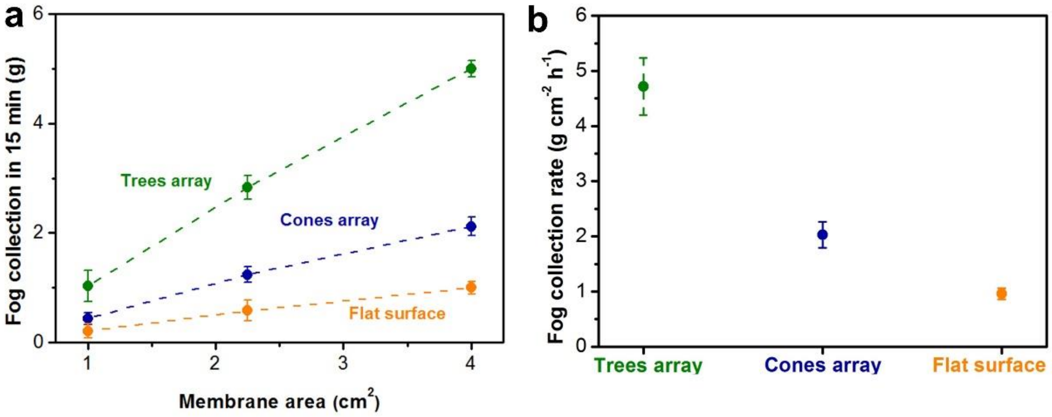

Supplementary Figure 11. (a) Fog collection rates of microstructured PVA/PPy gel membranes with different projected membrane areas. Their fog collection rate during steady states has a proportional relationship to the membrane area, as far as the entire membrane is covered by full fog flow. (b) Areal fog collection rate remained constant for each structure. The results demonstrate that fog collection rates of different gel structures can be normalized by their total surface area and thus the effects of other factors can be examined separately.

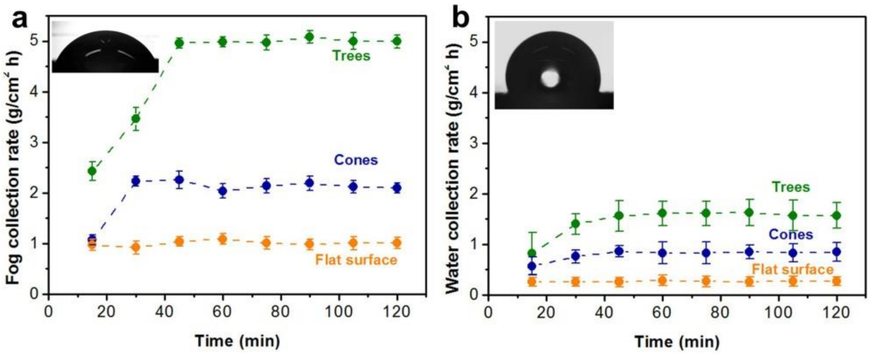

Supplementary Figure 12. Fog collection rates of (a) PVA/PPy gel membranes and (b) cured PR48 membranes with different microstructures. The insets show the contact angle tests of two materials. The hydrophobicity of surface can affect fog collection behavior[7,8]. Structured PR48 membranes show much worse fog collection performance than PVA/PPy gel membranes with same microstructures, demonstrating that hydrophilic nature of PVA/PPy hydrogel can benefit fog collection through facilitating fog droplets deposition.

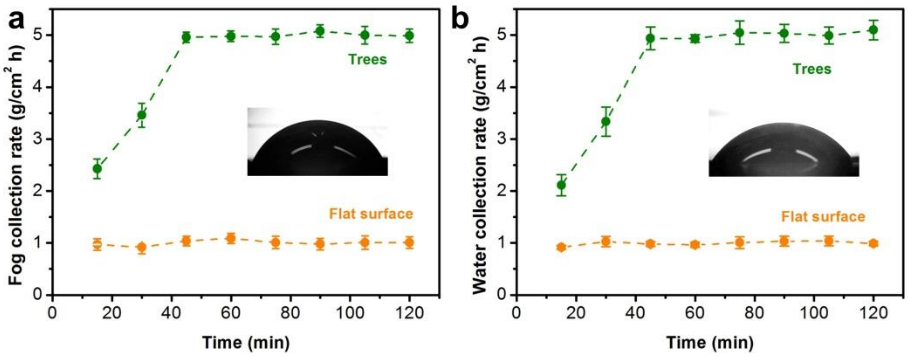

Supplementary Figure 13. (a) Fog collection rates of (a) PVA/PPy gel membranes and (b) pure PVA hydrogel membranes with different microstructures. The insets show the contact angle tests of two materials. The addition of PPy doesn't affect the fog collection behavior of micro-structured hydrogels. The reason could be that the PPy particles are firstly synthesized and then added to PVA solution for gelation. They are embedded in the PVA matrix, rather than on the gel surface.

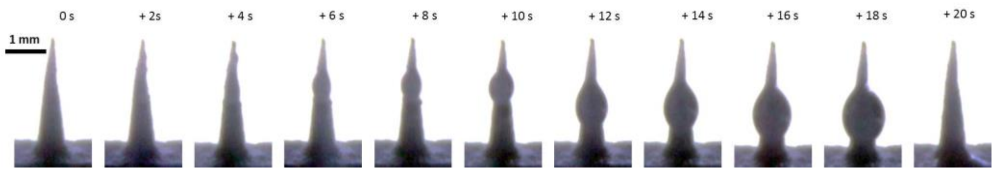

Supplementary Figure 14. Photos showing fog collection behavior of PVA/PPy gel micro-cone. After the fog flow is applied, tiny droplets deposit on the gel surface. From the 4th second, one major droplet forms due to coalescence of small droplets and it moves towards the base of cone. The droplet keeps growing by absorbing fog droplets in air and new deposited droplets on gel surface during its directional movement. After around 20 s, the droplet drains from the gel cone and the whole surface is refreshed.

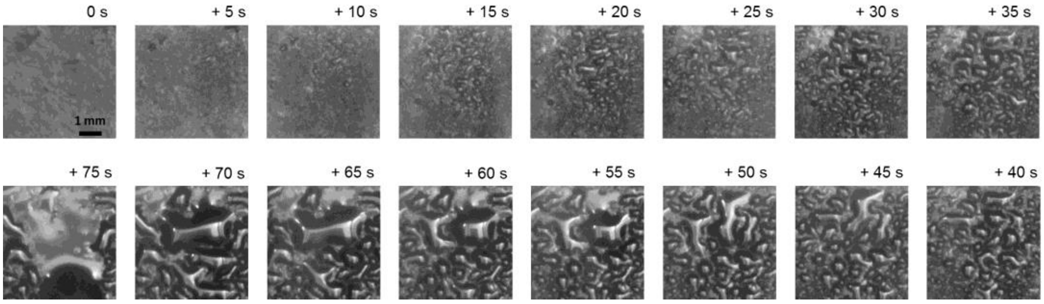

Supplementary Figure 15. Photos showing fog collection behavior of PVA/PPy gel flat surface. Tiny water droplets randomly deposit on the smooth gel surface after fog flow is applied. With continued deposition, the water drops increase their size through directly capturing drops in fog or coalescing with other drops nearby but without obvious transfer of mass center in either case. After 75 s, a large and heavy enough water droplet forms and drains off from the gel surface. The absence of quick regeneration of the fresh deposition sites in the overall process counts against the fog collection.

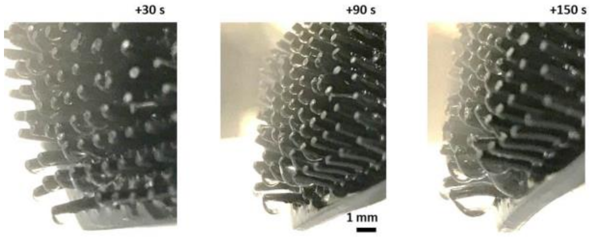

Supplementary Figure 16. Photos showing fog collection behavior of PVA/PPy gel cylinders. After the initial drop forms on the gel cylinder, the size of droplet keeps increasing with a much lower rate by absorbing water in fog flow. The droplet sticks on the gel surface without obvious movement of its mass center. After more than 2.5 min, the droplet falls from the gel cylinder when it becomes too large for the structure to support its weight. The sticking behavior leads to even worse fog collection performance of gel cylinders than that of flat surface.

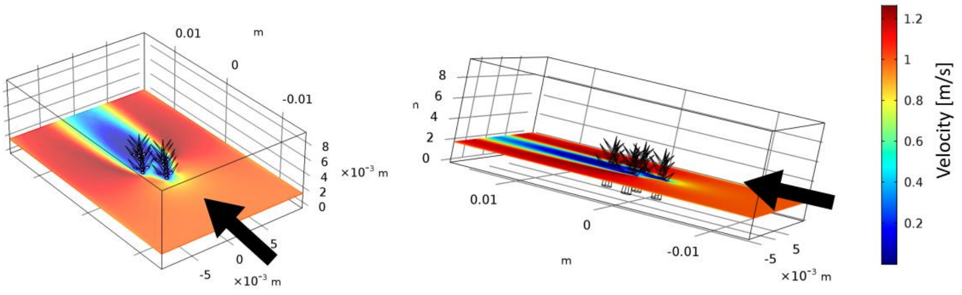

Supplementary Figure 17. Simulated flow field for a configuration with a reduced number of tree elements. The dynamics were modeled with the Reynolds-averaged Navier-Stokes (RANS) equations, with automatic wall treatment and default COMSOL flow parameters. Arrow corresponds to the inflow direction, with the boundary velocity of 1 m/s at the inlet. This qualitative result corroborates the assessment that the PVA/PPy gel micro-tree array disrupts and slows down the fog flow field.

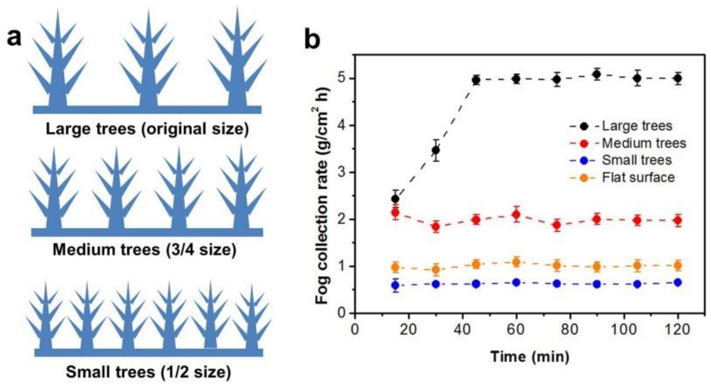

Supplementary Figure 18. (a) Schematic illustration of gel micro-tree arrays with different sizes (side view). The dimension (both height and bottom diameter) and inter-distance of gel trees are 3/4 (medium trees) and 1/2 (small trees) of original ones. (b) Fog collection rates of gel micro-tree arrays with different sizes in fog harvesting tests. The fog collection rate of PVA/PPy gel micro-tree arrays decreased significantly as the size of array reduced. The small trees array shows even worse fog collection ability than gel membrane with flat surface. This is caused by inefficient drainage of collected water to beaker. Due to reduced inter-space, the water droplets are trapped among gel micro-trees and can’t be drained to beaker efficiently.

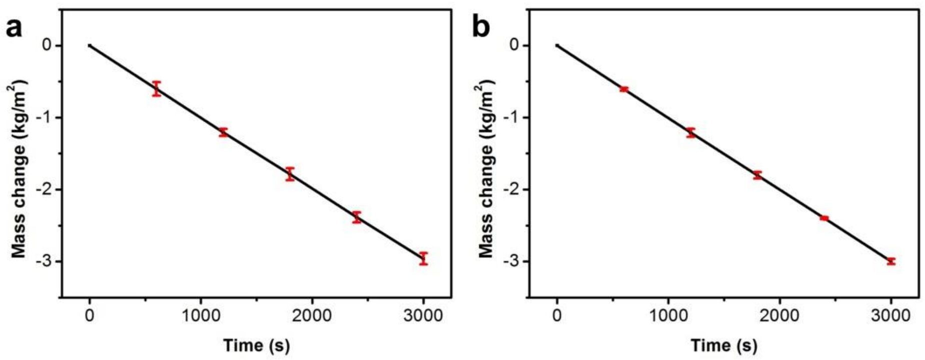

Supplementary Figure 19. Solar steam generation properties of PVA/PPy gel micro-tree arrays with (a) 3 cm × 3 cm and (b) 5 cm × 5 cm sizes.

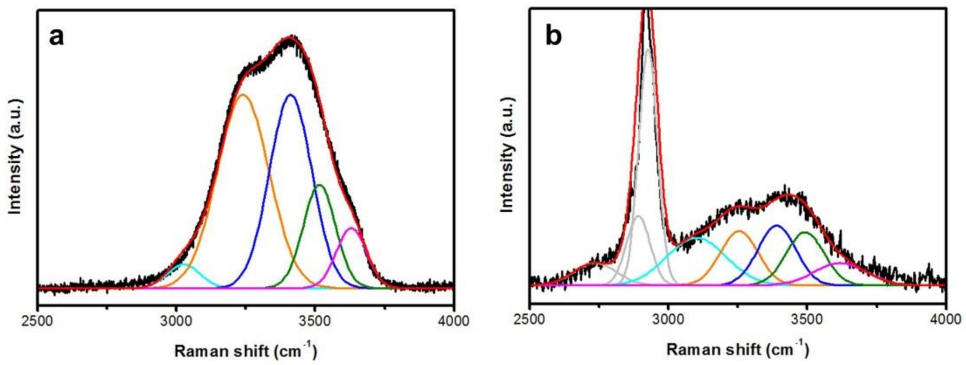

Supplementary Figure 20. Raman spectra and fitted peaks of (a) free water and (b) PVA/PPy hydrogel[9]. The peak around 3050 cm^{-1} (light blue) arises from the Fermi resonance between the overtone of the bending mode. The three peaks observed at 2735, 2875, and 2920 cm^{-1} (grey) in hydrogel spectra can be assigned to the C–H stretching mode of CH, the symmetric, and asymmetric stretching modes of CH2 on PVA chains, respectively. Four peaks at 3250, 3395, 3505, and 3610 cm^{-1} are observed in both spectra, which are related to water molecules. These peaks are classified as two types of modes: (1) Water molecules forming full hydrogen bonds with adjacent water molecules (the peaks at 3250 and 3395 cm^{-1}); (2) weakly or non-hydrogen-bonded water molecules in which the hydrogen bonds of the water molecules have been broken, in part or entirely (the peaks at 3505 and 3610 cm^{-1}). Within the band corresponding to the four hydrogen-bonded molecules, the peak at 3250 cm^{-1} is associated with the collective in-phase vibrations of all molecules in the aggregate, whereas the 3395 cm^{-1} peak is associated with vibration, which is not in-phase between the first and higher shell of neighboring molecules. The peaks at 3505 and 3610 cm^{-1} correspond to the symmetric and asymmetric stretching of the weakly hydrogen bonded water molecules, respectively. We calculated the molar ratio of weakly bonded water: fully bonded water in free water and PVA/PPy hydrogel,

which are 0.25:1 and 0.74:1, respectively. The weakly bonded water in free water can be attributed to the surface tension induced hydrogen bonds weakening. These results indicate that much water contained in PVA/PPy hydrogel is weakly bonded due to interactions with PVA chains, which reduces the water vaporization enthalpy and facilitates the water evaporation.

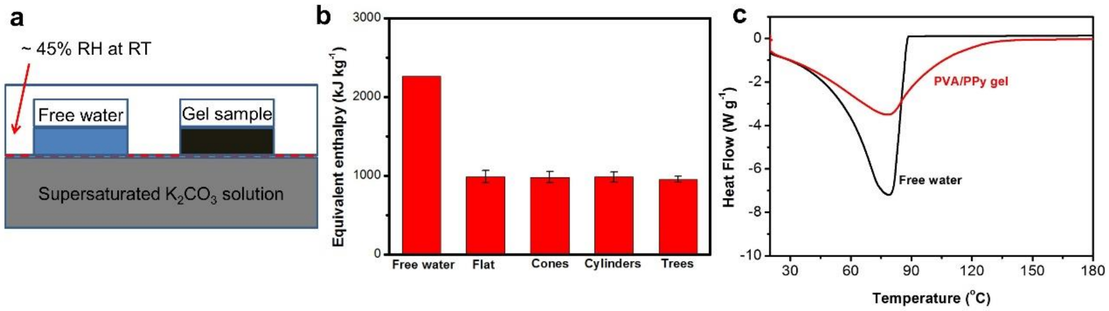

Supplementary Figure 21. (a) Schematic illustration of experimental set-up for equivalent vaporization enthalpy measurements. (b) Calculated equivalent vaporization enthalpy of water in microstructured PVA/PPy gels. (c) DSC measurements on free water and PVA/PPy hybrid gel. The measured enthalpy of free water is 2424 kJ kg^{-1}, which is very close to the theoretical value of 2450 kJ kg^{-1}. The water vaporization enthalpy in hybrid gel is 1735 kJ kg^{-1}. Note that the enthalpy values calculated from DSC are higher than those tested in evaporation experiments, since the DSC test and evaporation test present a full dehydration and slightly dehydration processes, respectively[1].

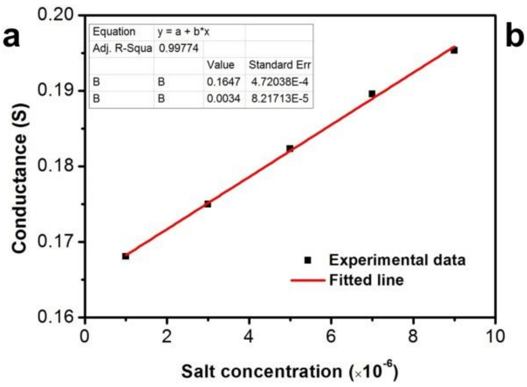

b

Salinity before purification (‰)	Average salinity after purification (‰)
35	0.00198
100	0.00618

Supplementary Figure 22. (a) Linear dependency of salinity and conductance of NaCl solution under 25 ºC. (b) The calculated salinity of purified water. Antifouling property has been demonstrated for hydrogel based solar steam generators in several studies[10,11]. The salt accumulation issue was not observed in our studies. The mechanism behind is that the hydrogel layer builds a salt equilibrium by water transport-induced salt ion absorption and diffusion-enabled salt ion discharge.

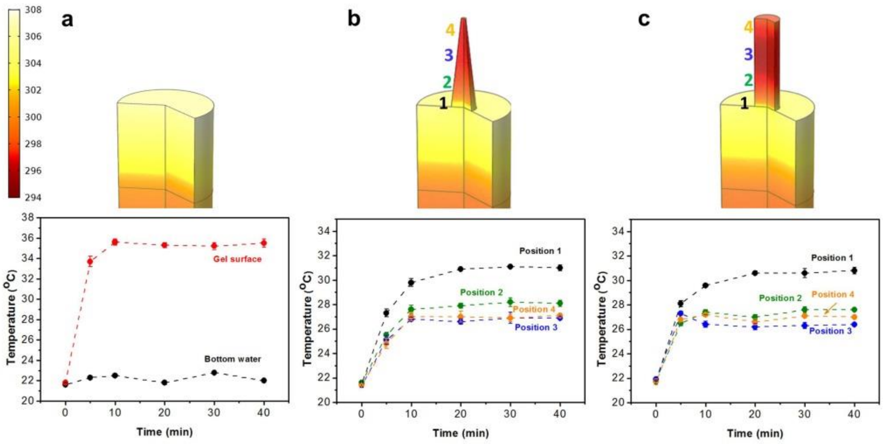

Supplementary Figure 23. Simulation and experimental measurements of surface temperature distribution of PVA/PPy gel structures: (a) flat surface, of which larger depth below surface is heated. This part of energy is wasted for heating water in gel matrix under evaporation surface rather than supporting vapor generation. (b) cone, which can absorb light through its entire surface. However, its titled surface towards normal light irradiation leads to lower light absorption by unit area. (c) cylinder, which shows reduced temperature in the middle section because this part of cylinder can't absorb sunlight directly. The surface of the base is the hottest for all geometries (a-b-c); for cones (b), the temperature decreases from the base towards the cone tip; for pillars (c), the temperature decreases along the pillar, but increases towards the base/tip. The experimentally measured temperatures are consistent with the observed trends in our qualitative model.

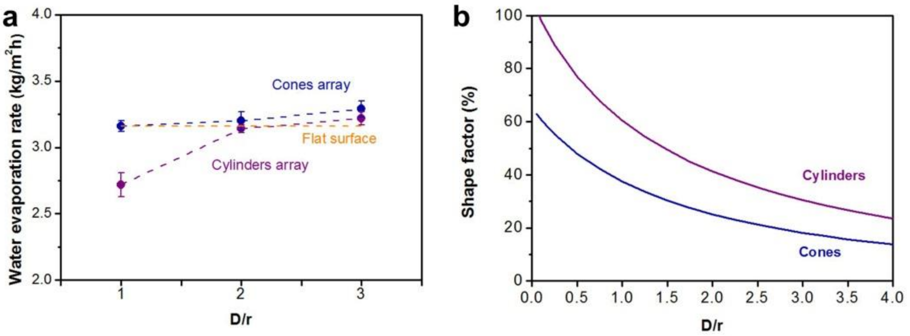

Supplementary Figure 24. (a) Measured solar vapor generation rates and (b) calculated shape factor of PVA/PPy gel cone and cylinder array with changed inter-distance. D is the inter-distance between two adjacent microstructures and r is the bottom radius of gel cone or cylinder. Geometric factor could affect the solar vapor generation performance of gel microstructure arrays by affecting the escape of generated vapor flow. Owing to its tapered shape, the conical structure has the potential to more effectively guide the vapor flow away from the evaporating surface.

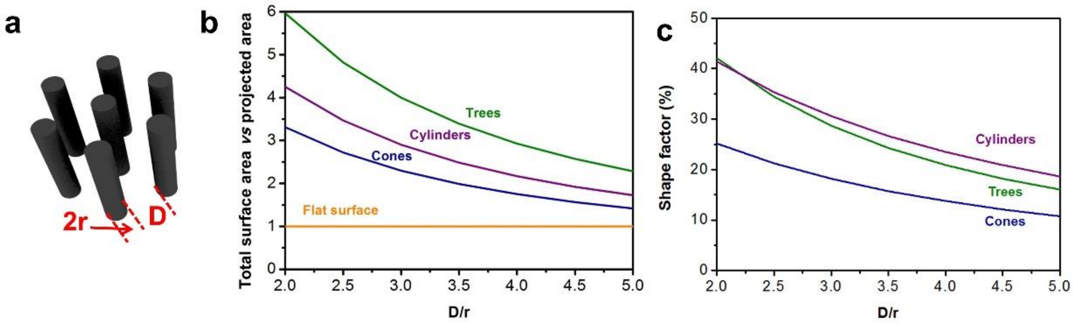

Supplementary Figure 25. (a) Schematic illustration of calculation of D/r value. (b) Calculation of total surface area vs projected area for gel membranes with different microstructures arrays. (c) Calculation of the shape factor for different microstructure arrays. As the D/r value decreases and the gel micro-structures are more closely packed, the total surface area of micro-trees array increases more rapidly than other micro-structures arrays but its shape factor remains comparable to that of cylinders array. Thus the gel micro-trees array enables a larger evaporation area for a comparable shape factor, indicating that the generated vapor could still efficiently escape when the surface area for evaporation is greatly increased.

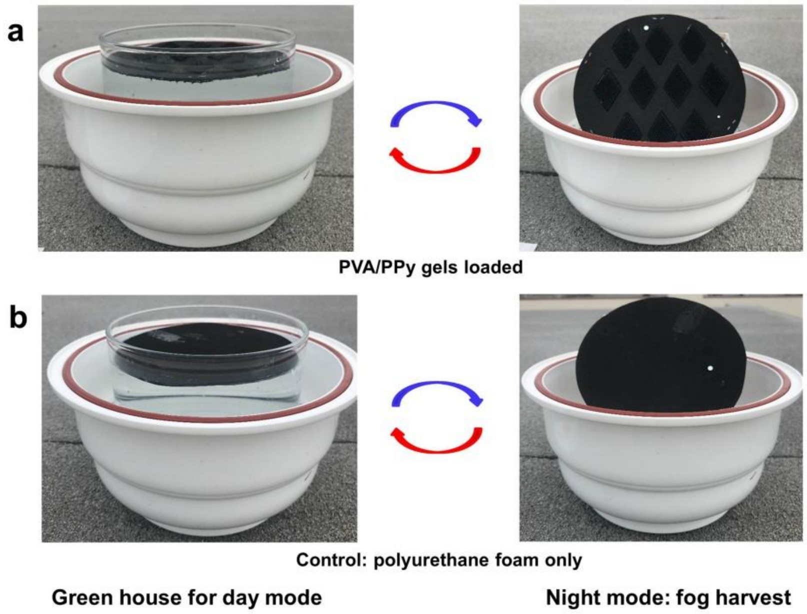

Supplementary Figure 26. (a) Photos showing rooftop tests set-up for solar driven water purification during daytime and fog harvesting during night. The prototype was placed on the roof of one Caltech building. 10 pieces of PVA/PPy gels (~55 cm^2 in total) were held by a supporting structure which was made by a 1-mm thick layer of polyurethane foam and nylon mesh. During daytime (8:00 am to 20:00 pm), the gel loaded structure floated on a basin of brine water (3.5 wt% NaCl solution) and covered the whole water surface. A transparent cover was applied on the prototype. During night (20:00 pm to next day's 8:00 am), the transparent cover was removed and the gels were supported to face air flow with 45 degree tilted. (b) Set-up of control experiment using polyurethane foam as control sample. From the desiccator with gel samples, we collected 180 to 200 ml water after 12 h solar vapor evaporation. From the control experiment, we usually got 5 to 20 ml water, which is subtracted.

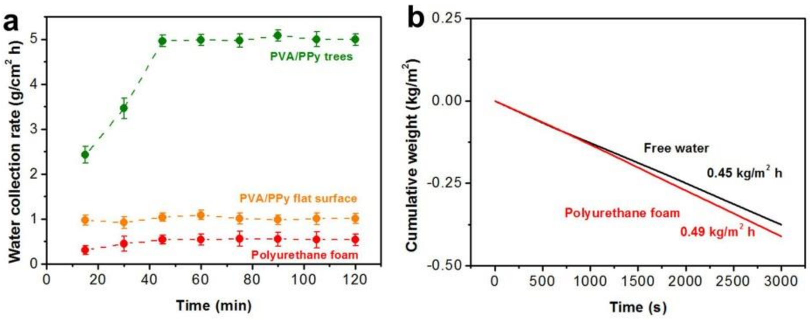

Supplementary Figure 27. (a) Fog collection and (b) solar vapor generation performance of polyurethane foam tested in lab. The polyurethane foam shows low fog collection rate of ~0.5 g cm^{-2} h^{-1} and solar vapor generation rate of ~0.49 kg m^{-2} h^{-1}.

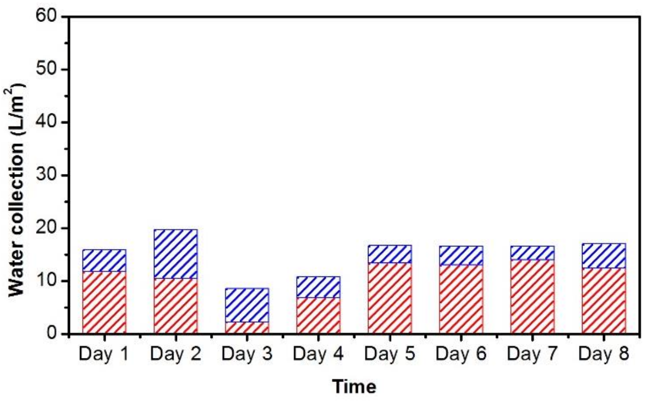

Supplementary Figure 28. Daily water collection per square meter of total water surface during rooftop tests. Red: water collected during daytime (8 am to 8 pm); blue: water collected during nighttime (8 pm to next day's 8 am). The daily water collection rate is much lower because about half of the water surface was covered by the passive supporting structure made by PU foam.

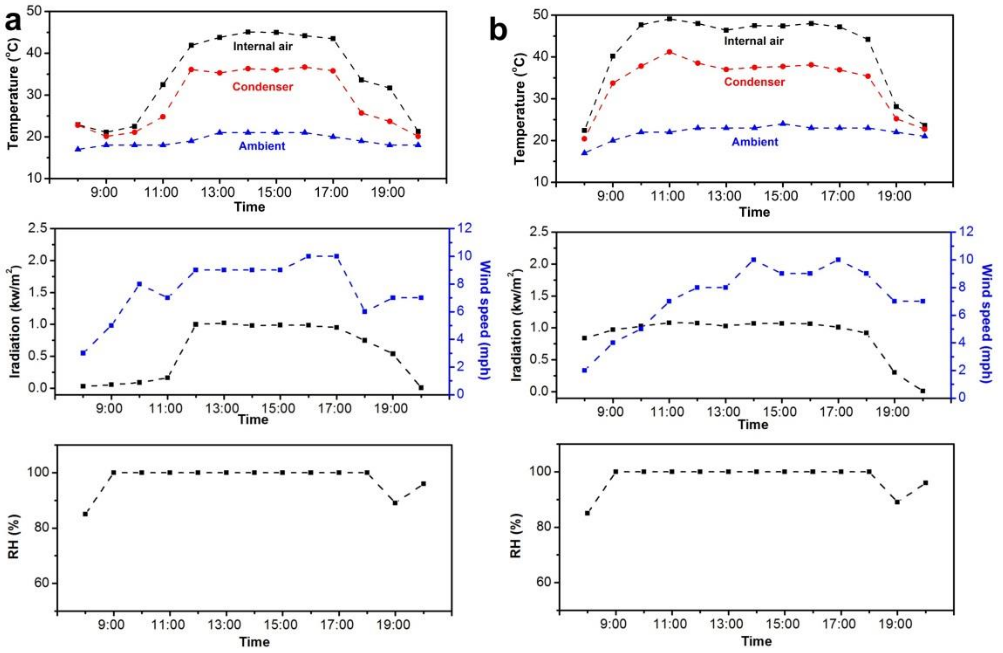

Supplementary Figure 29. Solar irradiation intensity, temperature inside the desiccator, temperature on the desiccator surface, ambient temperature (from local weather report), wind velocity (from local weather report), and relative humidity inside the desiccator recorded during two rooftop tests: (a) test on 06/26/2020 and (b) test on 07/10/2020. When the gel samples are fully working under 1 sun irradiation, the temperature on the desiccator surface is around 10 °C lower than the internal temperature and 15 to 20 °C higher than the ambient temperature. The wind velocity is from 2 to 10 mph. These factors help on efficient water condensation. The condensation area in our outdoor system is more than 30 times larger than that of gel samples. It is reported that when the temperature of water vapor in the solar water-purification system is around 40 °C[12], the upper bound of the condensation rate of a convective condenser is less than 0.1 kg m^{-2} h^{-1}. Consider the condensation area in our case is more than 30 times larger than evaporation area, the system could support efficient condensation for our gel samples.

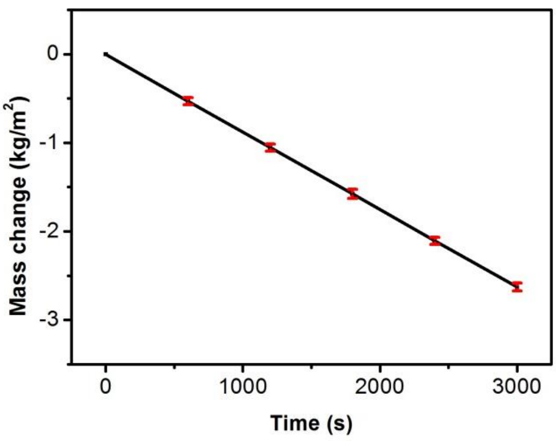

Supplementary Figure 30. Solar steam generation properties of PVA/PPy gel micro-tree arrays in a closed system (relative humidity ~100%). The performance decrease caused by high humidity can be solved or relieved by system optimization.

.

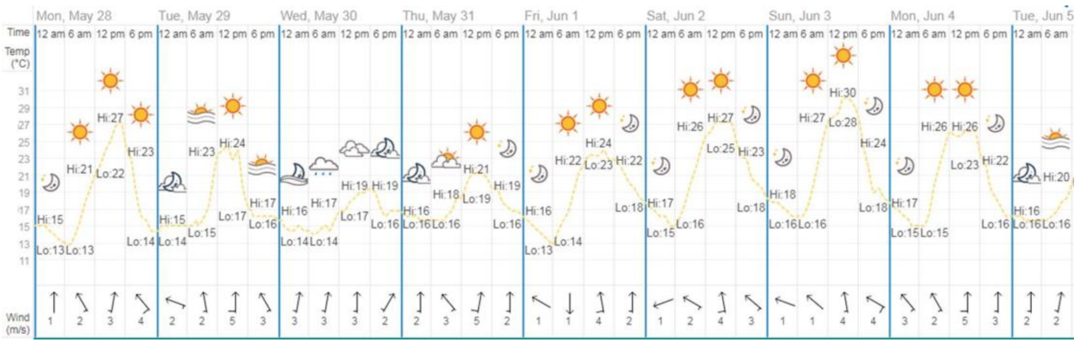

Supplementary Figure 31. Weather conditions of Pasadena from 05/28/2018 to 06/04/2018[13].

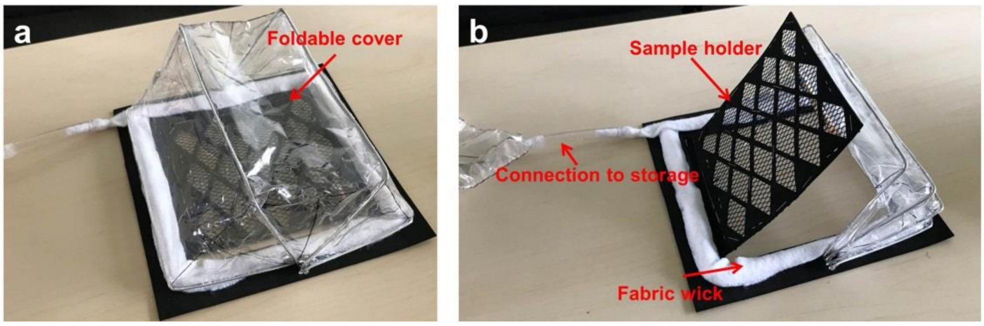

Supplementary Figure 32. (a) Day mode and (b) night mode of floating prototype for water collection. During daytime, the condensation structure is closed and the generated vapor re-condenses on it, which is collected by fabric wicks and transported to water storage. During night, the cover is open and the gel samples can be supported to face the fog flow. The collected water is guided to fabric wicks and stored in floating bag.

Supplementary Table 1. The calculated salinity of water during in lab fog collection test. A PVA/PPy gel sample was used to evaporate NaCl solution with salinity of 100 under one Sun irradiation for 6 h. Then a lab fog collection test was applied on this gel sample for 6 h. The artificial fog was generated by adding dilute NaCl solution into the humidifier. The salinity of collected water was measured and compared to that of water in the humidifier. This solar evaporation and fog collection cycle was repeated for 10 times using the same gel sample.

Fog collection test #	Average salinity of collected water (‰)	Average salinity of water in humidifier (‰)
1	0.00126	0.00119
2	0.00148	0.00122
3	0.00137	0.00138
4	0.00119	0.00121
5	0.00122	0.00135
6	0.00130	0.00117
7	0.00134	0.00129
8	0.00125	0.00109
9	0.00113	0.00136
10	0.00117	0.00125

Supplementary Table 2. Daytime water collection in outdoor prototypes using PU foam with and without holes

Daytime water collection	PU foam w/ holes	PU foam w/o holes
Day 1	13.6 mL	14.2 mL
Day 2	15.2 mL	14.7 mL
Day 3	6.8 mL	6.5 mL
Day 4	11.3 mL	10.5 mL
Day 5	8.8 mL	9.1 mL
Average	11.14 mL	11.00 mL

Supplementary References

1. Zhao, F., Zhou, X. Y., Shi, Y., Qian, X., Alexander, M., Zhao, X. P., Mendez, S., Yang, R. G., Qu, L. T. & Yu, G. H. Highly efficient solar vapour generation via hierarchically nanostructured gels. *Nat. Nanotech.* **13**, 489-495 (2018).
2. Ju, J., Yao, X., Yang, S., Wang, L., Sun, R. Z., He, Y. X., & Jiang, L. Cactus Stem Inspired Cone-Arrayed Surfaces for Efficient Fog Collection. *Adv. Funct. Mater.* **24**, 6933–6938 (2014).
3. Ju, J. *et al.* Cactus Stem Inspired Cone-Arrayed Surfaces for Efficient Fog Collection. *Adv. Funct. Mater.* **24**, 6933-6938 (2014).
4. Lecoq, L., Flick, D. & Laguerre, O. Study of the water evaporation rate on stainless steel plate in controlled conditions. *Int. J. Therm. Sci.* **111**, 450-462 (2017).

5. El-Samadony, Y. A. F., El-Maghlany, W. M. & Kabeel, A. E. Influence of glass cover inclination angle on radiation heat transfer rate within stepped solar still. *Desalination* **384**, 68-77 (2016).
6. Ni, G. *et al.* A salt-rejecting floating solar still for low-cost desalination. *Energy Environ, Sci.* **11**, 1510-1519 (2018).
7. Dai, X., Sun, N., Nielsen, S. O., Stogin, B. B., Wang, J., Yang, S. K. & Wong, T. K., Hydrophilic directional slippery rough surfaces for water harvesting. *Sci. Adv.* **4**, eaaq0919 (2018).
8. Bai, H. *et al.* Efficient Water Collection on Integrative Bioinspired Surfaces with Star-Shaped Wettability Patterns. *Adv. Mater.* **26**, 5025-5030 (2014).
9. Kudo, K. *et al.* Structural changes of water in poly(vinyl alcohol) hydrogel during dehydration. *J. Chem. Phys.* **140**, 044909 (2014).
10. Zhou, X. *et al.* A Hydrogel-based Antifouling Solar Evaporator for Highly Efficient Water Desalination. *Energy Environ. Sci.* **11**, 1985-1992 (2018).
11. Guo, Y. *et al.* Biomass-Derived Hybrid Hydrogel Evaporators for Cost-Effective Solar Water Purification. *Adv. Mater.* **32**, 1907061 (2020)
12. Zhou, M. *et al.* Accelerating vapor condensation with daytime radiative cooling. *Proc. SPIE 11121, New Concepts in Solar and Thermal Radiation Conversion II*, 1112107 (2019).
13. https://www.timeanddate.com/weather/usa/pasadena-ca/historic.
