# Supplementary material for: All-day fresh water harvesting by microstructured hydrogel membranes
Source: Nat Commun. 2021 May 14;12:2797. doi: 10.1038/s41467-021-23174-0 (PMC8121874; doi:10.1038/s41467-021-23174-0)
Supplement: Supplementary file 3 — Description of Additional Supplementary Files [file 41467_2021_23174_MOESM3_ESM.pdf]

Description of Additional Supplementary Files

File Name: Supplementary Movie 1
Description: Fog collection and transportation on hydrogel micro-trees.
